# Medicine

**Published:** 1893-03

**Authors:** William C. Krauss


### Progress in Medical Science.

### MEDICINE.

By WILLIAM C. KRAUSS, M. D.

### THE VALUE OF TRIONAL AS AN HYPNOTIC.

Dr. Brie, Bonn. (*Neurologische Centralblatt*, December 15, 1892.) Trional is described as a powder consisting of tablets of a melting point of 76° C., readily soluble in alcohol and ether, but less soluble in water, requiring 320 parts of water at ordinary temperature. Trional belongs to the same chemical family as sulfonal and tetronal, but containing three ethyl groups instead of two as in sulfonal, and four as in tetronal. Its chemical composition is a diethyl sulfon methylmethan, and was first described by Kast and Baumann. The drug was given in fifteen to twenty-five grain doses, fifteen to forty-five minutes before retiring, and in those cases in which pure insomnia existed, sleep followed in from fifteen minutes to one and one-half hours after administration. Dr. Brie used it chiefly in mental diseases, dividing his cases into four classes. The first class included mild forms of melancholia and hypochondria; eleven cases were under treatment, and in all, seven to nine hours' sleep followed. The second class included four cases of agitated melancholia; these cases had been previously treated with opium, chloral, etc., without any favorable results. The first two cases were given thirty grains of trional with happy effect; the third patient was obliged to take forty grains, and the fourth patient was given fifteen grains.

The third group included the maniacal cases and paretics; ten cases were treated with but one failure, and that because of stomach irritation, necessitating abandonment of the drug. In these cases other hypnotics had been tried, such as chloral, paraldehyd, sulfonal, and tetronal, but the results were not as satisfactory.

The fourth class included paranoia and the hallucinations; eight patients were treated and all were successful. Besides these mental diseases—insomnia, due to overwork, worry, excluding pain, trional gave refreshing sleep. Trional differs from sulfonal in that the latter takes effect later and has an accumulative action; from tetronal, in that tetronal takes effect quicker, but the effect wears off sooner.

As a rule there are no disagreeable after-effects. (The translator has employed trional in a few cases of insomnia with excellent results, ten grains of the drug producing good, refreshing sleep.)

---

### A NEW SIGN IN THE DIAGNOSIS OF INTRA-CRANIAL AFFECTIONS.

IN THE January number of the *Anales del Circulo Medico Argentin*, Dr. Masi has an interesting article describing a new diagnostic symptom. The great importance of thoracic vibrations—their absence or exaggeration is appreciated in detecting diseases of the thoracic cavity. The same vibrations which occur during articulation are conveyed to the base of the skull, and thence to the vertex, where they can be detected as readily as over the thorax. Apply the palm of the hand to the head and one to the thorax, and the vocal fremitus will be as distinct to the one hand as to the other. If this sign, through its variations, can be utilized to help in the diagnosis of intra-thoracic affections, cannot it be likewise utilized in the diagnosis of intra-cranial affections? This is the question which the author tried to solve. A patient was brought to the hospital, who, through injury, sustained a fracture at the base of the skull with ramifications as far as the upper part of the temporal regions on both sides. All the common signs of fracture of the base, such as bleeding and escape of arachnoid fluid from the ears and nose, etc., were present. Over the left temporal region there was found to be depression of about two centimeters in diameter. The patient rallied, and on the eighth day, as the doctor was examining the head, the patient spoke and a distinct fremitus was perceived over the whole head, except over the depressed bone in the left temporal region. This was noticed by members of the hospital staff and regarded as a strange phenomenon, which would, perhaps, enable one to apply it to other brain and skull lesions. In a normal head, therefore, the vibrations should be distinctly perceived over all portions of the skull, but in those cases where there is increased pressure, such as tumors, abscesses, and hemorrhages, or where there is thickening of the dura or adhesions between the brain and the skull through the dura, or fractures, absence of this vibration would be expected. The author describes the sign as Cranial Vibrations, and awaits further corroboration of his discovery.

### THE RELATION OF GENITAL IRRITATION, IN THE MALE AND FEMALE, TO NERVOUS AND MENTAL DISEASES.

The above was the title of an interesting paper read by Dr. L. C. Gray, of New York, at the recent meeting of the New York State Medical Society.

A careful review of the history of genital irritation was first given, dating back to the belief of Hippocrates, (which has been the source of the modern doctrines upon the subject,) and this was followed by a discussion, in some detail, of the literature which sprang up after the publication of Stanley's paper, in 1833, as well as the further revival of the question by Sayre, in 1870.

Dr. Gray then went on to say that there was no proof that genital irritation was capable of causing any of the organic diseases of the nervous system, but that it might possibly act as an exciting or aggravating factor of certain lesser nervous diseases. In mental diseases, however, relief of genital irritation has proved, in his opinion, to be a much more valuable method of treatment than it seemed to be in the lesser nervous affections. He called attention, however, to the fact that there were two great classes of mental diseases—namely, the organic insanities and those which are known as the psychoneuroses—and that, so far as was yet known, it was only in the latter that relief of the genital irritation was of any value. He then gave the histories of several classes of insanity in which operations upon the female genitalia had given prompt and permanent relief. He was not, however, willing to believe that all the relief was due to the removal of the genital irritation, because simple etherization had sometimes worked almost as well, and, in one case, had done quite as much as the operation itself would have effected. He furthermore called attention to the fact, that if the operation upon the female genitalia was of any value at all in the relief of mental disease, it should only be done at a proper stage of convalescence. His conclusions were as follows :

1. That there is no proof that genital irritation, in the male or female, can cause nervous or mental disease, except in the predisposed individual.

2. That the proof is not yet absolute that genital irritation can produce nervous or mental disease, even in the predisposed individual.

3. That there is undoubted proof that relief of genital disease, in the male or female, will often relieve certain nervous diseases, such as migraine, hysteria, epilepsy, simple nervousness, and hallucinatory insanity.—*Medical and Surgical Reporter, February 11, 1893.*

---

ELECTRICITY IN THE TREATMENT OF DIARRHEA OF CHOLERA.

IN THE *Progrès Médical* for January 28, 1893, Dr. Arslan reports upon the use of electricity in the various forms of diarrhea, particularly of cholera, as practised in the wards of Dr. Jules Simon, at the Höpital des Enfants-Malades. Having observed that the faradic current applied directly to the abdominal walls succeeded in stopping the diarrhea in the first few cases tried, it was used in all cases, even in intestinal tuberculosis, which is generally rebellious to all forms of treatment, with gratifying success. In the diarrheas of dysentery and ulcerated entero-colitis did the method fail.

The simplest form of a faradic battery is sufficient, and the current should be strong enough to produce visible contractions of the abdominal muscles. Both poles should be placed upon the abdomen for intervals of one to two minutes. As a rule, after the third or fourth treatment the diarrhea ceases, accompanied with amelioration of the other symptoms, as fever, vomiting, anorrhexia, etc. In some cases a single application is sufficient. No inconveniences are observed by the patient and the current is well borne. During the past Summer (July, 1892,) the writer had occasion to treat two cases of cholera at the above mentioned hospital, one a child aged nine years, the other twelve. In both of these cases the diarrheas ceased after the first application. In some thirty cases treated in the same way, the results were in every way favorable, although several applications were necessary. No other treatment was instituted—save hygiene and strict attention to diet—and the favorable results may, without contradiction, be attributed to the faradic current.

---

THE SYRUP OF HYDRIODIC ACID AND ITS USES.

DR. REYNOLD W. WILCOX, Professor of Clinical Medicine, at the New York Post-Graduate School and Hospital, comes to the following conclusions regarding hydriodic acid :

1. In syrup of hydriodic acid we have a palatable and efficient method of administering iodine.

2. The dosage is under control. We can saturate the patient with iodine, yet iodism can be avoided.

3. The field of usefulness is greater than that of iodide of potash or any other alkaline iodine.

4. The failure of administration in former times was due to the impossibility of making a high-class pharmaceutical which should be of sufficient iodine strength and absolutely permanent; here the recent improvements in manufacture are of the greatest importance.—*The Post-Graduate, February, 1893.*

---

### NOCTURNAL INCONTINENCE OF URINE AND PHIMOSIS.

In an article on the above subject by Dr. E. Loumeau, in the January number of *Annales de la Policlinique de Bordeaux*, the writer divides the different varieties of incontinence under five heads:

1. Incontinence of urine of purely psychopathic origin. (Theory of T. L. Petit.)
2. Incontinence of urine due to vesical irritability. (Theory of Trousseau.)
3. Incontinence of urine due to a faulty contractility of the sphincter vesicæ, or to anesthesia of the urethra. (Theory of Guyon.)
4. Incontinence of urine due to paralysis of the bladder and sphincter.
5. Incontinence of urine due to epilesy.

Under the second head—vesical irritability—the author mentions the following causes: (1) Peripheral irritation, such as phimosis, atresia of the meatus, hypospadias, oxyurides, hemorrhoids, fissures, etc. (2) Exaggerated reflex excitability of the cord. The author regards phimosis as the most prolific agent in this classification. Trousseau appears to have been the first to call attention to this fact in 1860, and since then Forni, Tuffier, Beard, Bouisson, Duplay, Schwartz, Birger, and many others, have called especial attention to this form of enuresis.

---

### TREATMENT OF MIGRAINE.

Prof. Hare (*College and Clinical Record*) recommended the following treatment for migraine: During the intermissions give cannabis indica in small doses, (gr. $\frac{1}{4}$ of the extract,) and when the patient feels that an attack is threatening give a large dose (gr. j.) with tincture of gelseminum, gtts. xx., and usually the attack may be aborted.

### ANGEIO-NEUROTIC EDEMA.

In the *American Journal of the Medical Sciences* for December, 1892, Dr. Joseph Collins, of New York, has made a thorough and complete study of angeio-neurotic edema—examining critically all the published cases, and, as a result, has drawn the following conclusions :

The conclusions concerning the pathology of this disease are :

1. That there exists a variety of edema attended by such striking characteristics of its own that we are justified in referring its origin to the nervous system.

2. The seat of the manifestation of the lesion is probably in those vessels and lymphatics which pass through the corium to the subdermal tissues.

3. It is probable that although the lesions or the irritants on which the disease is dependent may attack other parts of the system, yet the result directly appears through the sympathetic system.

4. Evidence concerning the bearing of trophic influences in the production of the disease cannot be produced. But when trophic changes do occur, they are more plausibly attributed to the changes brought about by the oft-recurring edema, *per se*, than to influences exerted through the nervous system as true trophoneuroses.

5. It is quite possible that in the future its causation may be attributed and shown to be dependent upon products manufactured and ordinarily disposed of within the system, but which, acted on by sinister influences, either inherited or acquired, result in the temporary disturbance of the vasomotor system, which is manifested in various parts of the body, depending, as does the analagous condition of the distribution of blushing and flushing, upon structural peculiarities, either central or peripheral, or upon inherent predilections.

6. This condition has a close relationship to the many edemas spoken of, and also a family relation with many of the arthropathies as yet not well understood, but known to be directly caused through the agency of the nervous system.

7. It must be admitted, from clinical evidence, that the affection in question has a family relation with other vaso-motor neuroses, such as exophthalmic goitre and urticaria.

